# Prediction of Metal Ion–Binding Sites in Proteins Using the Fragment Transformation Method

**DOI:** 10.1371/journal.pone.0039252

**Published:** 2012-06-18

**Authors:** Chih-Hao Lu, Yu-Feng Lin, Jau-Ji Lin, Chin-Sheng Yu

**Affiliations:** 1 Graduate Institute of Molecular Systems Biomedicine, China Medical University, Taichung, Taiwan; 2 Institute of Bioinformatics and Systems Biology, National Chiao Tung University, Hsinchu, Taiwan; 3 Bioinformatics Program, Taiwan International Graduate Program, Institute of Information Science, Academia Sinica, Taipei, Taiwan; 4 Institute of Biomedical Informatics, National Yang-Ming University, Taipei, Taiwan; 5 Department of Information Engineering and Computer Science, Feng Chia University, Taichung, Taiwan; 6 Master's Program in Biomedical Informatics and Biomedical Engineering, Feng Chia University, Taichung, Taiwan; Institute of Enzymology of the Hungarian Academy of Science, Hungary

## Abstract

The structure of a protein determines its function and its interactions with other factors. Regions of proteins that interact with ligands, substrates, and/or other proteins, tend to be conserved both in sequence and structure, and the residues involved are usually in close spatial proximity. More than 70,000 protein structures are currently found in the Protein Data Bank, and approximately one-third contain metal ions essential for function. Identifying and characterizing metal ion–binding sites experimentally is time-consuming and costly. Many computational methods have been developed to identify metal ion–binding sites, and most use only sequence information. For the work reported herein, we developed a method that uses sequence and structural information to predict the residues in metal ion–binding sites. Six types of metal ion–binding templates– those involving Ca^2+^, Cu^2+^, Fe^3+^, Mg^2+^, Mn^2+^, and Zn^2+^–were constructed using the residues within 3.5 Å of the center of the metal ion. Using the fragment transformation method, we then compared known metal ion–binding sites with the templates to assess the accuracy of our method. Our method achieved an overall 94.6 % accuracy with a true positive rate of 60.5 % at a 5 % false positive rate and therefore constitutes a significant improvement in metal-binding site prediction.

## Introduction

The structure of a protein determines its function and its interaction(s) with other components, e.g., other proteins and cofactors, including metal ions. Approximately one-third of all proteins bind at least one metal ion [1], [2], [3], and many different types of metal ion–binding proteins are found in humans [4], [5]. Metal ions help stabilize protein structure, may induce a conformational change upon binding, and/or participate in catalysis. Metal ions found in proteins include those of the alkali metals, alkaline earth metals and transition metals, with the most common being sodium and potassium ions, calcium and magnesium ions, and iron, manganese, copper and zinc ions, respectively. For the metal ion–binding proteins found in the Protein Data Bank (PDB http://www.rcsb.org/pdb/), ∼66 % contain transition metal ions, ∼37 % contain alkaline earth metal ions, and ∼6 % contain alkali metal ions [6].

In humans, hemoglobin transports oxygen in the blood from the lungs to peripheral tissues. Hemoglobin contains four heme groups that reversibly bind Fe^2+^. Fe^2+^coordinates four heme nitrogens and, reversibly, one oxygen. In the absence of an oxygen, a water molecule is bound. Urease, expressed by the Gram-negative microaerophilic bacterium *Helicobacter pylori*, requires Ni^2+^ for its function. Urease hydrolyses urea into carbon dioxide and ammonia to produce an alkaline environment that protects the bacterium from acidic gastric juice during its infection of the stomach. Thus, in both prokaryotes and eukaryotes, metal ion–binding proteins are extensively involved in many different biochemical reactions. Identifying metal ion–binding sites is, therefore, key to understanding the functional mechanisms of metal ion–binding proteins.

Experimentally, metal ion–binding proteins are identified and/or characterized using nuclear magnetic resonance spectroscopy [7], gel electrophoresis [8], metal-affinity column chromatography [9], electrophoretic mobility shift assay [9], absorbance spectroscopy [10], and mass spectrometry [8]. Most of these methods require complex steps and specialized equipment, making them unsuitable for unknown targets. There is considerable demand, therefore, for other ways to identify metal ion–binding sites. Computational methods have been used to identify metal ion–binding sites, e.g., support vector machines [6], [11], [12], neural networks [6], [13], the FoldX force field [14], the CHED algorithm [15], [16], graph theory and geometry algorithms [17], [18]. Some of these methods use only sequence information [6], [11], [12], whereas others use both sequence and structure information [17], [18]. However these previous attempts to predict metal ion–binding sites have often had low sensitivities; clearly, predictive accuracy must be improved.

On average, the members of the Structural Genomics Initiative solve 20 new protein structures each week. Currently, the PDB contains more than 70,000 protein structures. In general the regions in proteins that interacts with ligands, substrates, or other proteins tends to be structurally conserved [19] and the residues involved are in close spatial proximity even though they may be distant in sequence. Such residues constitute ∼ 10–30 % of a protein sequence [20], [21], [22]. The residues that most often bind metal ions are CYS, HIS, GLU and ASP [23], [24] because the atoms of their polar or charged side chains can coordinate metal ions. For the work reported herein, we used the fragment transformation method [25] to identify residues in proteins that bind Ca^2+^, Cu^2+^, Fe^3+^, Mg^2+^, Mn^2+^, or Zn^2+^. This method combines sequence and structural information contained within spatially local fragments. Given that the three-dimensional structure and residue type are often conserved, similar binding regions can be found by comparing the types of residues and their relative locations with those of computationally constructed metal ion–binding residue templates.

**Figure 1:**
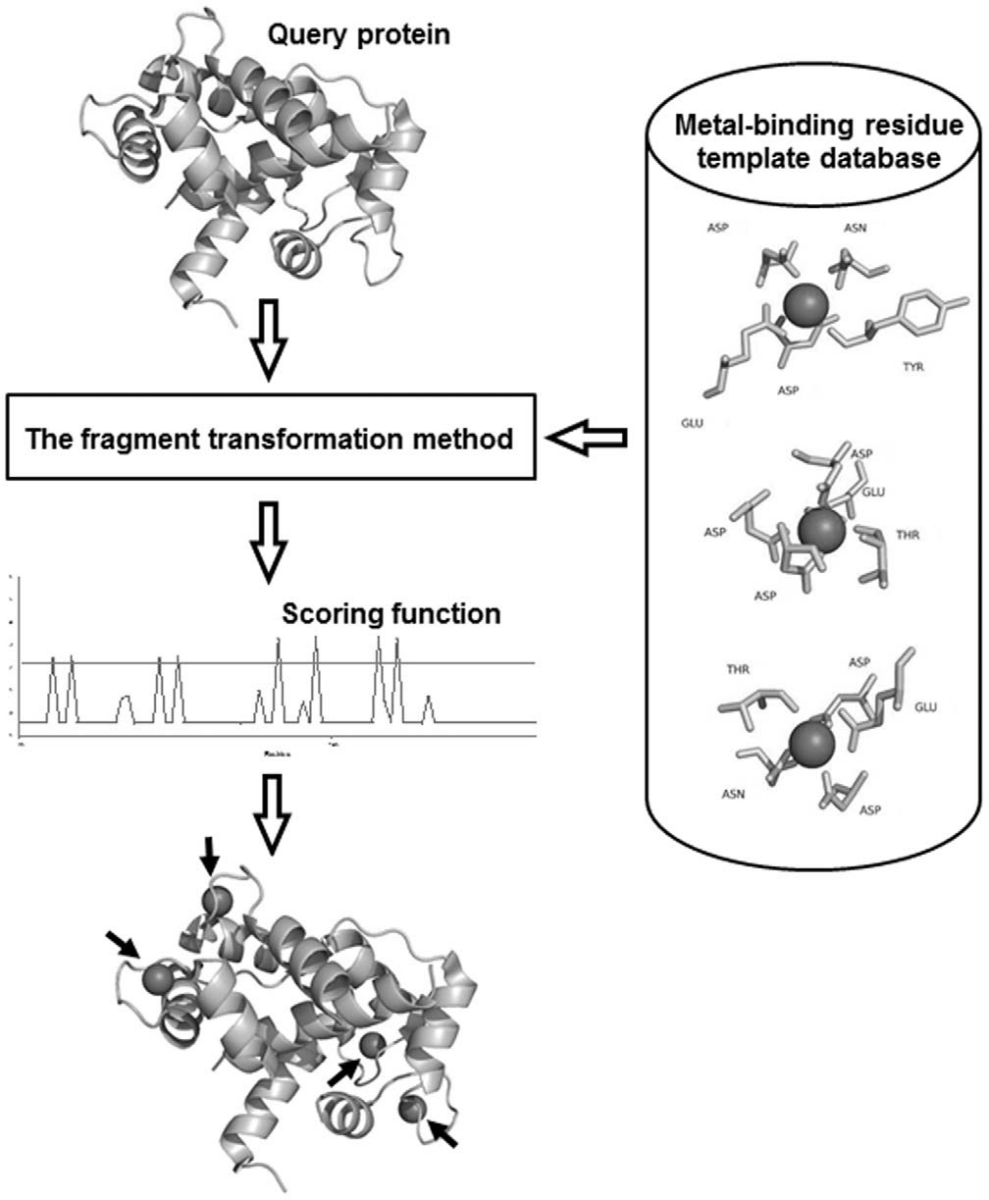
Schematic of the metal ion–binding prediction method.

## Methods

### Overview

First, the structures of known metal ion–binding proteins were extracted from the PDB. Next, a database containing metal ion–binding sites templates was constructed. Each template included residues at least partially within 3.5 Å of the metal ion center. The structure of the protein being queried for a metal ion–binding site (query protein) was then compared with each template using a “leave-one-out" comparison method. The fragment transformation method [25] attempts to structurally align fragments of the query protein and the metal ion–binding residue template. After each comparison, each residue in the query protein was assigned an alignment score that is composed of two functions for the evaluation of sequence and structure conservation. The sequence similarity is calculated by using the BLOSUM62 substitution matrix [26], and the structure similarity is taken by measuring the root mean square deviation (RMSD) of the Cα carbons of the local structural alignments. Residues that score above the assigned alignment-score threshold are predicted to bind metal ions. This method is illustrated in Figure 1.

**Figure 2:**
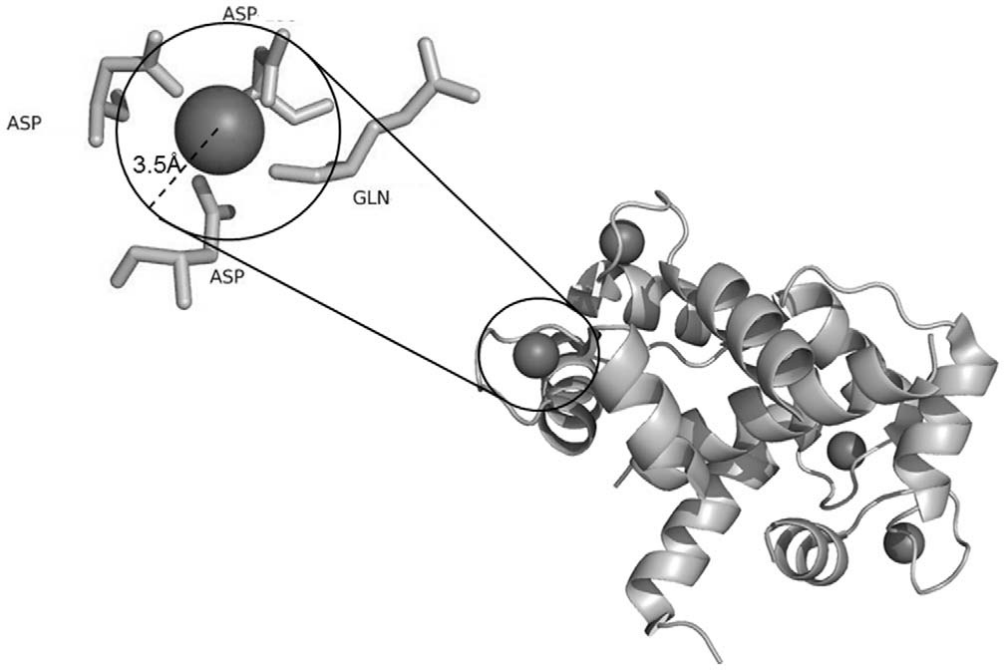
Metal ion–binding residues. All residues at least partially within 3.5 Å of a metal ion are defined as metal ion–binding residues.

**Table 1:** The types and number of metal ion–binding polypeptides and metal ion–binding residue template.

Metal ion	Number of polypeptides	Number of templates
Ca^2+^	273	407
Cu^2+^	47	74
Fe^3+^	51	77
Mg^2+^	256	209
Mn^2+^	110	144
Zn^2+^	372	499
Total	1109	1410

**Figure 3:**
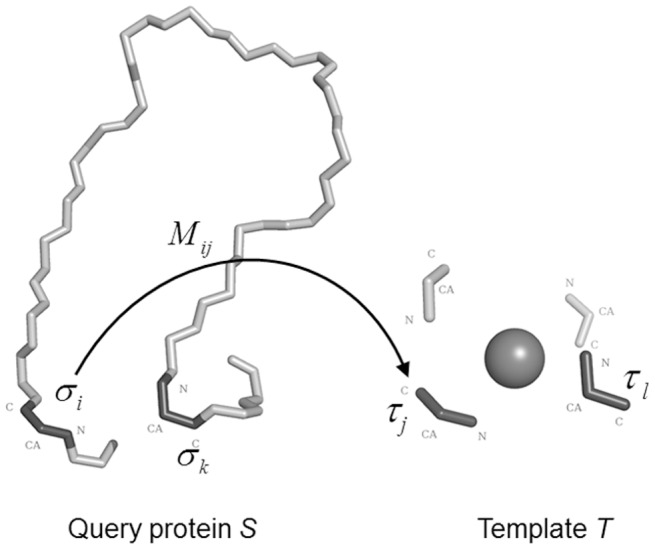
The fragment transformation method. *σ_i_* and *σ_k_* are two arbitrary triplet units in the query protein *S*, and *τ_j_* and *τ_l_* are two arbitrary triplet units in the template *T*. In the illustration, the triplet *σ_i_* is transformed onto *τ_j_* via application of the transformation matrix *M_i,j_*.

### Dataset containing the metal ion–binding proteins

The proteins in the final dataset were extracted from the PDB and contain at least one Ca^2+^, Cu^2+^, Fe^3+^, Mg^2+^, Mn^2+^, or Zn^2+^ ion. At the time of our study, approximately one-fourth of all PDB entries (20094 of 77294 proteins) contained a metal ion(s). The following criteria were applied to these proteins as filters. If the structures did not contain any polypeptide chain, those structures were excluded. For proteins containing more than one polypeptide chains, we included only the chains with residues involved in metal ion–binding. The length of the polypeptide chain was required to be more than 50 residues. DNA and/or RNA components were removed, leaving only the polypeptide chain.

**Figure 4:**
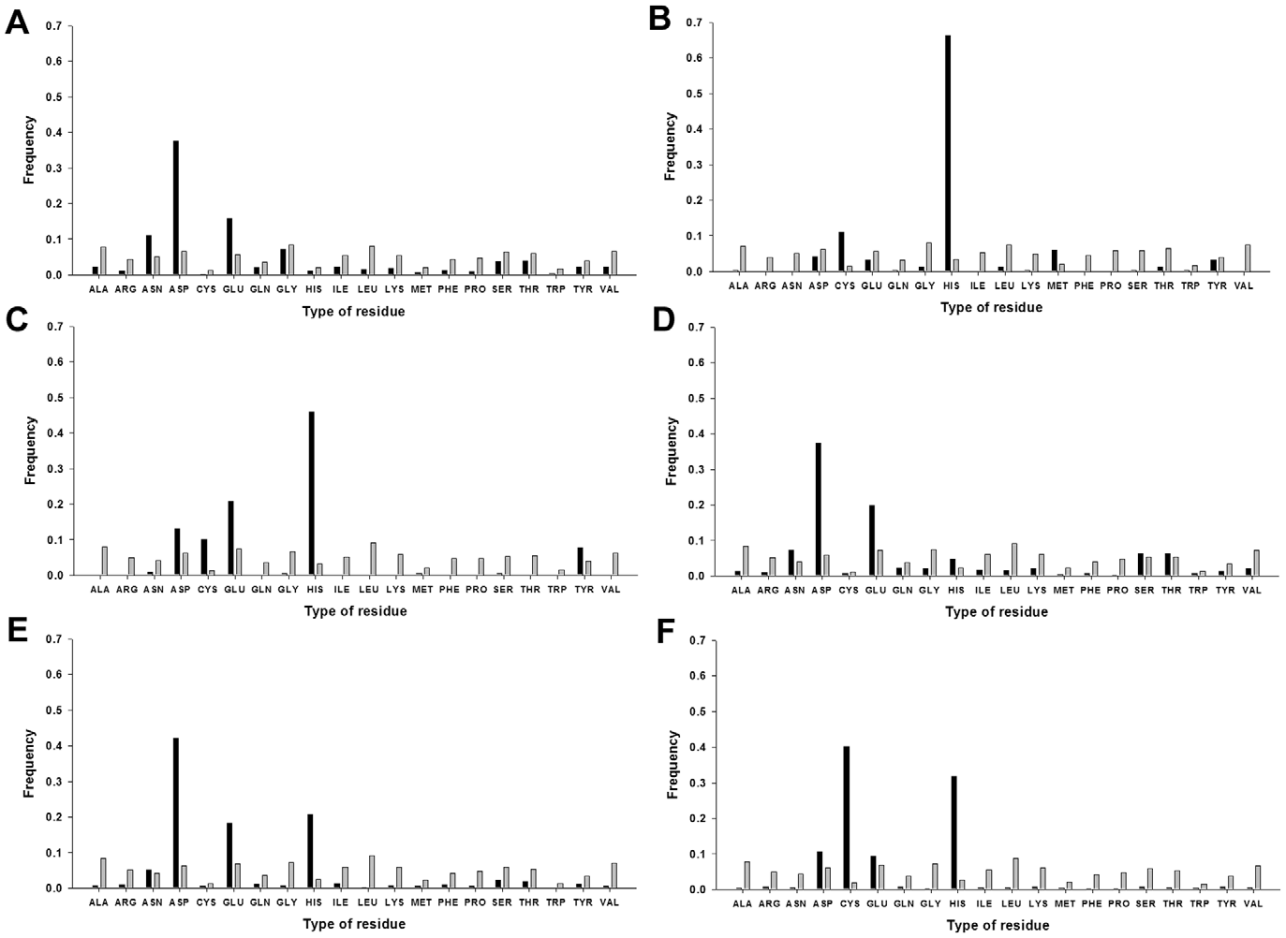
Frequency of each amino acid in the metal ion–binding sites. Frequencies of each amino acid in a given type of metal ion–binding site (black) and in the corresponding protein (grey). **A**, Ca^2+^. **B**, Cu^2+^. **C**, Fe^3+^. **D**, Mg^2+^. **E**, Mn^2+^. **F**, Zn^2+^. For this study, 1,109 metal ion–binding polypeptides were used and the metal ion–binding sites were defined as residues partially within 3.5 Å of the metal ion.

**Figure 5:**
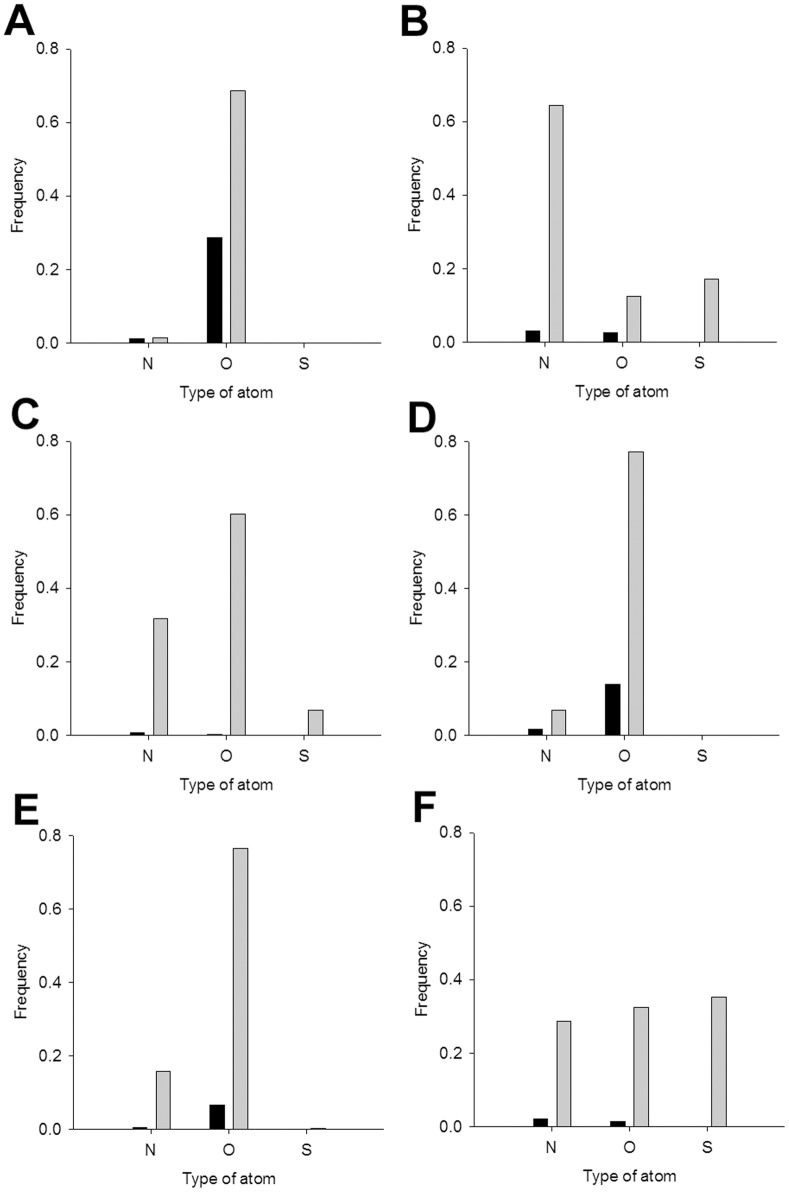
Frequency of atom types in the metal ion–binding sites. Frequency of each type of atom in the backbone (black) and in the side chain (grey). **A**, Ca^2+^. **B**, Cu^2+^. **C**, Fe^3+^. **D**, Mg^2+^. **E**, Mn^2+^. **F**, Zn^2+^.

To ensure that many different types of proteins were included in the dataset, proteins were grouped according to their superfamily by SCOP (version 1.67) [27]. Proteins that could not be classified by in this manner were removed. Finally, BLASTClust, in the standalone BLAST package (version 2.2.10) [28], was used to align the sequences in a pairwise fashion so that the remaining proteins could be sorted into groups that had sequence identities ≥ 25%. This step was performed to remove the redundant structures from the dataset because sequences with at least 25 % identity usually have similar conformations. For each cluster we retained the first entry as representative of the cluster. The final dataset is composed of 1,109 polypeptides representing 361 SCOP superfamilies.

**Figure 6:**
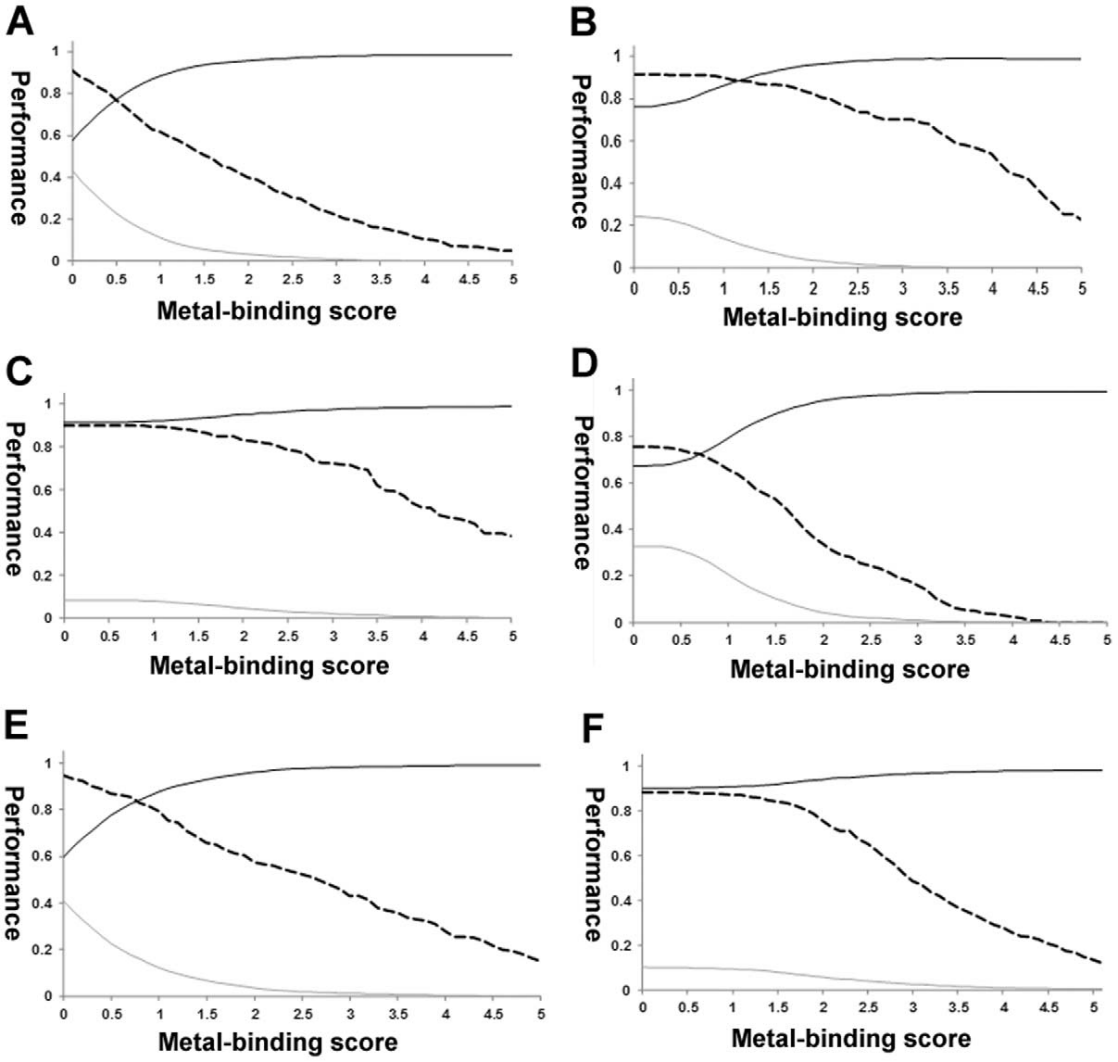
Metal ion–binding site prediction as functions of the metal ion–binding threshold scores. Accuracy (black solid line), true positive rate (dashed line), and false positive rate (grey line) as functions of the threshold values. **A**, Ca^2+^. **B**, Cu^2+^. **C**, Fe^3+^. **D**, Mg^2+^. **E**, Mn^2+^. **F**, Zn^2+^.

### Metal ion–binding residue templates

Figure 2 shows an example of a local structure containing metal ion–binding residues, *i.e.*, those at least partially within 3.5 Å of a metal ion center as judged by their PDB coordinates. To be considered as a template, a site needed contain more than two metal ion–binding residues. In total, 1,410 templates were generated from the 1,109 polypeptides. Table 1 list the statistics for each kind of metal ion–binding polypeptide and metal ion–binding template.

**Figure 7:**
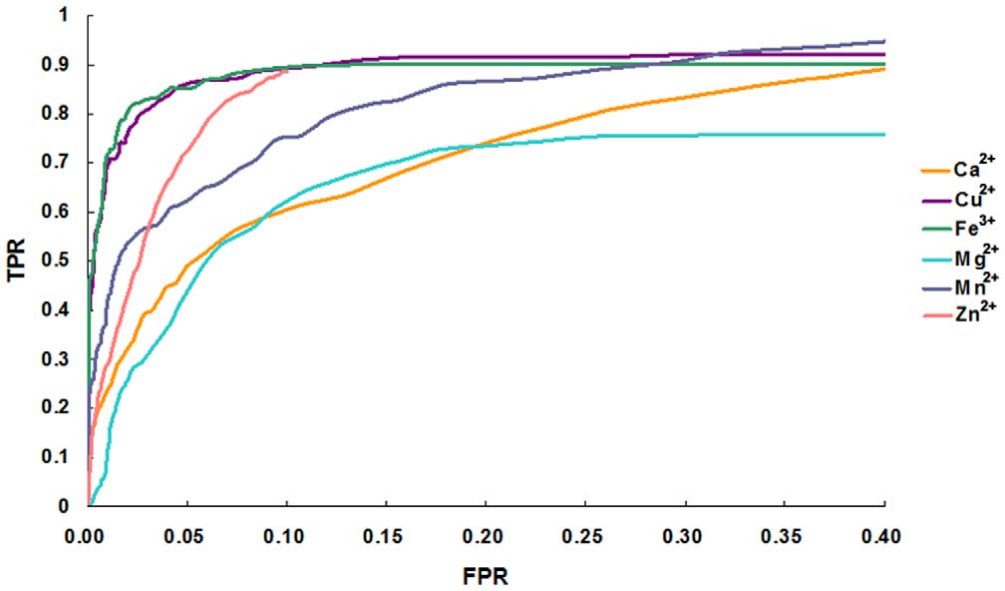
Receiver operating characteristic curves generated from the metal ion–binding site prediction. The performance of the method was assessed by measuring the areas under the receiver operating characteristic curves. The *x* axis reports the false positive rate (FPR), and the *y *axis reports the true positive rate (TPR).

**Table 2:** Comparison of the results for the fragment transformation and the artificial neural network methods.

Metal ion	ANN		This work	
	Accuracy (%)	TPR (%)	Accuracy (%)	TPR (%)
Ca^2+^	93.9	30.4	94.1	48.9
Cu^2+^	94.9	36.2	94.9	85.6
Fe^3+^	94.9	48.8	94.9	85.4
Mg^2+^	94.2	32.4	94.6	37.0
Mn^2+^	94.7	38.8	95.0	61.4
Zn^2+^	94.6	47.8	94.8	71.1
Overall	94.5	39.1	94.6	60.5

### The fragment transformation method

In general, the fragment transformation method [25] aligns similar local fragments that contain residues that are discontinuous in sequence but spatially close; for our study, the method was modified to align metal ion–binding residues. The fragment transformation method treats each binding residue as an individual unit. The structural unit used to align the query protein and the templates is a triplet formed by the backbone 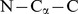 atoms of a given residue. *S* denotes the query protein of length *m*, *T* denote template of *n* residues. The 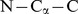 triplets of *S* and *T* be given by (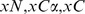) and (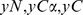) respectively, where *x* and *y* are the PDB coordinates for that atom. *S* and *T* can therefore be expressed in terms of the triplets as 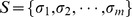 and 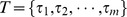, where
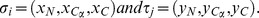



Note that the information contained in the peptide bonds preceding and following a residue is not used, meaning that 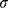 and 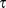 are not representative of the backbone torsion angles, φ and ϕ, which require the coordinates of 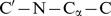 and 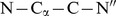, respectively, where C’ is the carbonyl carbon preceding the residue and N’’ is the amide nitrogen of the next residue. Thus, the fragment unit do not contain information concerning the torsion angles.

A matrix of dimensions 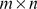 is then constructed for the residues of 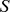 and 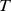 as:
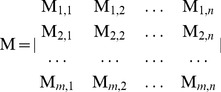
(1)where the element 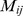 is a rigid-body transformation matrix that transforms the triplet 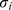 to 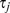, *i.e.*, 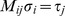. Each transformation matrix 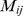 contains three rotations around and three translations along the *x*, *y* and *z* Cartesian axes (Figure 3).

**Figure 8:**
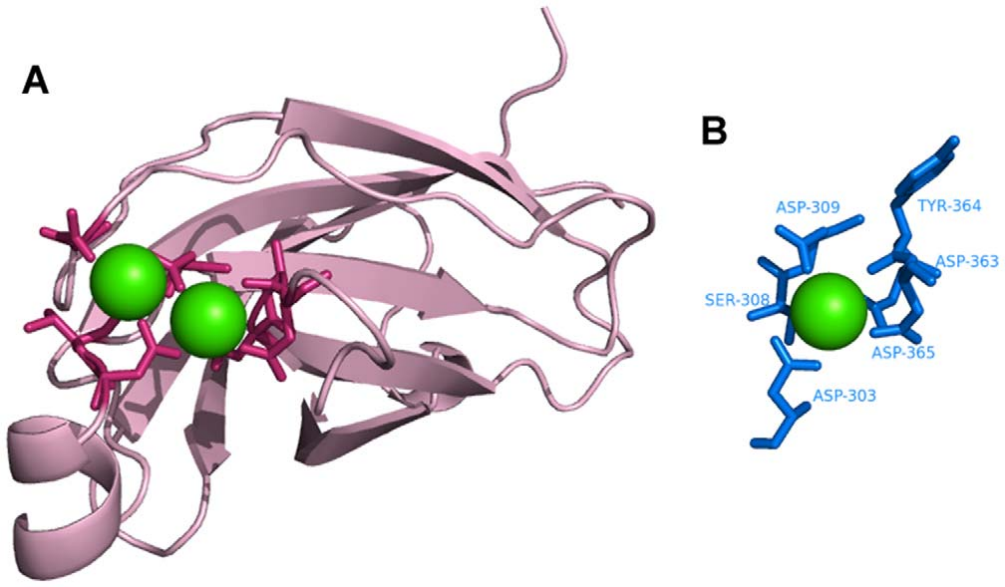
Identification of Ca^2+^–binding sites. **A**. human cytosolic phospholipase A2 (PDB ID∶1RLW) as the query protein. **B**. Template constructed from chain A of synaptotagmin I C2B-domain (PDB ID∶1K5W).

**Figure 9:**
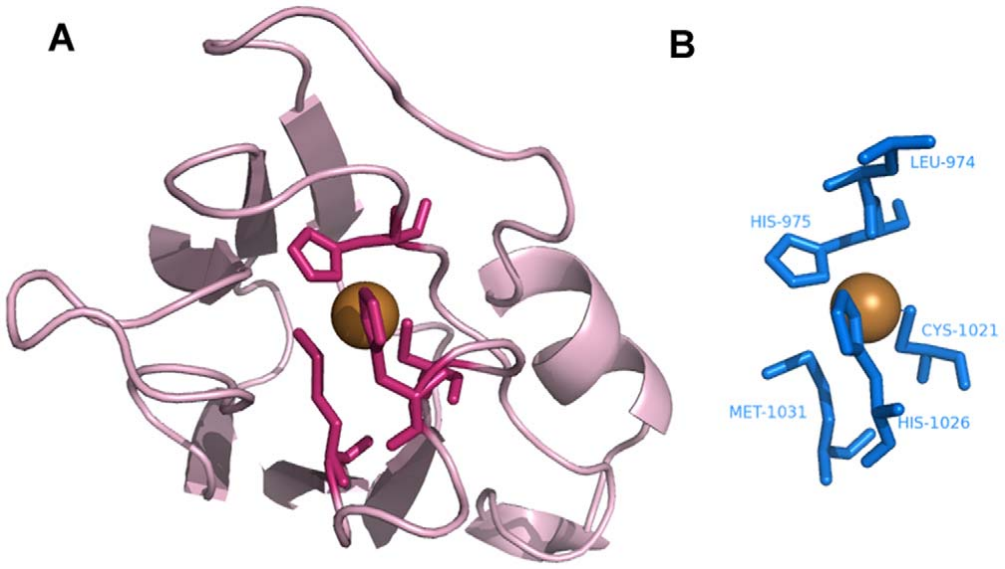
Identification of Cu^2+^–binding sites. A . Chain A of plastocyanin from the cyanobacterium *Phormidium laminosum* (PDB ID∶1BAW) as the query protein. **B**. Template constructed from plastocyanin (PDB ID∶1KCW).

### Performing triplet clustering


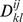
, defined as the Cartesian distance between the target 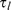 and the transformed triplet 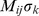, provides a measure of how similar the orientation of the triplet pairs 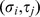 and 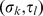 is, which allows us to cluster the triplet fragments using the single-linkage algorithm [29] as follows. If for two triplet pairs,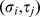 and 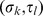, 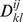 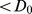, and 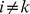 and 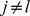, then the triplets are clustered. Let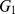 and 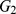 be two clusters, the first containing 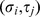 and 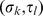 and the second containing 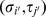 and 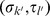. If 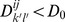, then 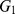 and 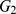 are merged to form a new cluster 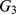, where 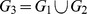. The procedures are carried out iteratively until no new clusters can be formed. For each final cluster 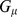, we obtain the aligned substructure pair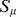 and 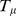, where 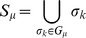 and 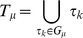.

**Figure 10:**
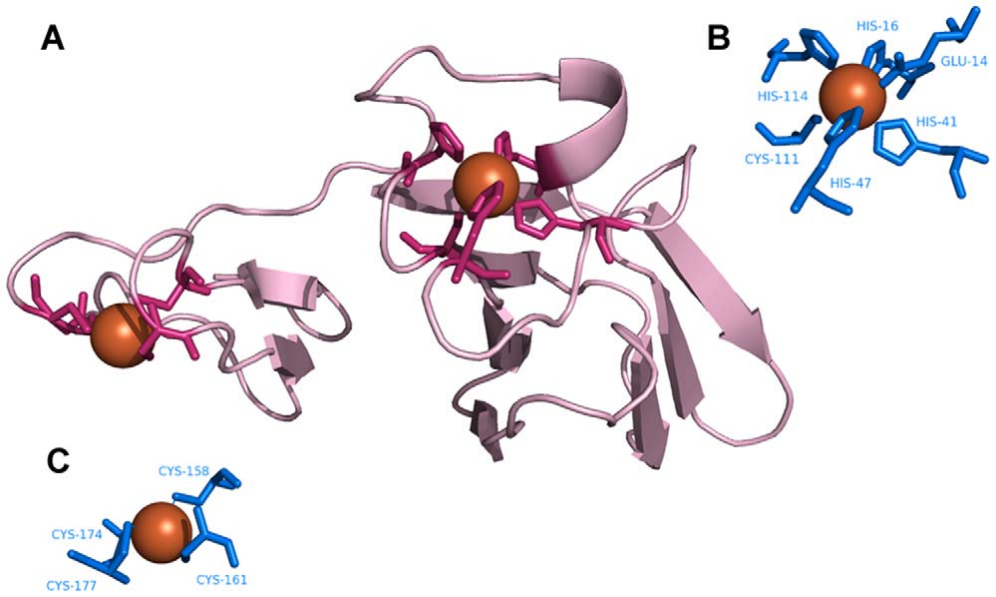
Identification of Fe^3+^–binding sites. **A**. Desulfoferrodoxin (PDB ID∶1DFX) as the query protein. **B**, **C.** Templates constructed from (**B**) chain A of superoxide reductase (PDB ID∶1DO6∶A) and (**C**) chain A of rubrerythrin (PDB ID∶1B71).

**Figure 11:**
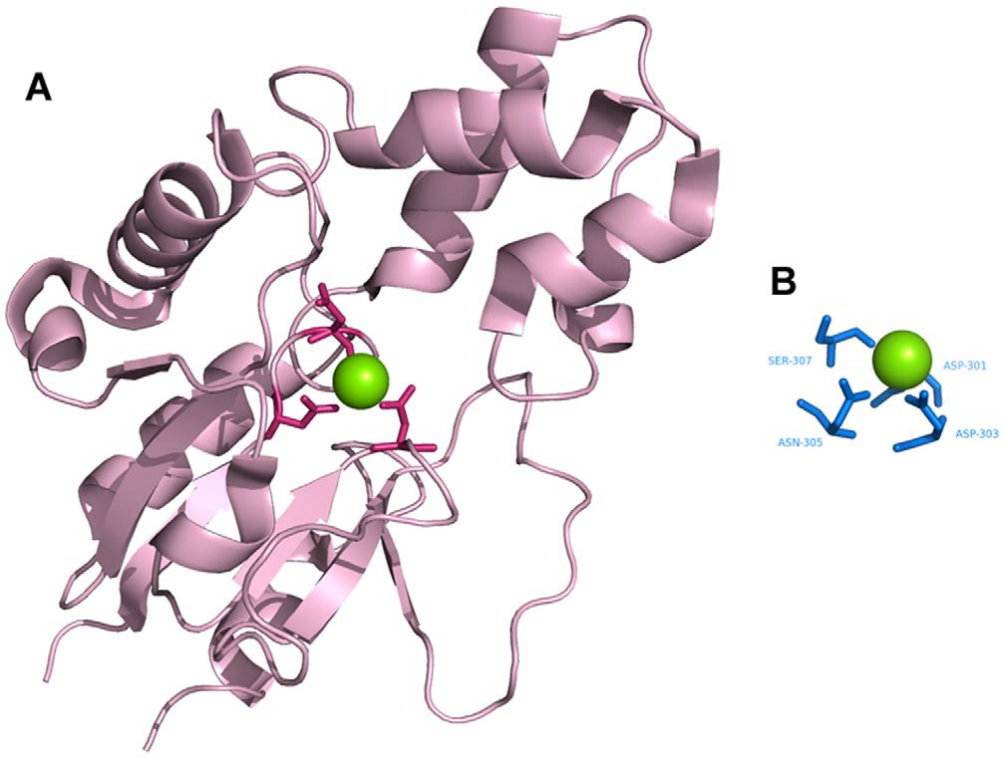
Identification of Mg^2+^–binding sites. **A**. Chain A of human mitochondrial deoxyribonucleotidase (PDB ID∶1MH9) as the query protein. **B**. Template constructed from chain B of transglutaminase 3 (PDB ID∶1NUG).

### Scoring function

The metal ion–binding score, 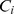, for each residue *i*, is defined as
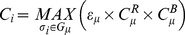
(2)where 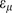 is the number of triplets of 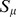, *i.e.*, the aligned residues of the query structure. The alignment scores 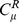, 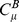 are defined as:



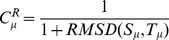
(3)


and



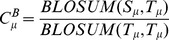
(4)where 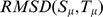 is the root mean square deviation of all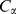 atoms between 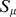 and 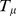;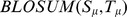 is the sequence alignment score between 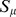 and 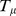, calculated using the BLOSUM62 [26] substitution matrix, and 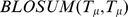 is the maximum sequence alignment score of 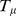. The value of 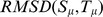 should be less than 3 Å, and 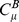 should be greater than 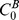 which can be adjusted to obtain the best result for each type of metal ion. Finally, the normalized metal ion–binding score, 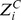, is calculated as:



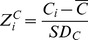
(5)where 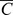 and 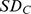 denote the mean and the standard deviation, respectively, of the metal ion–binding score.

**Figure 12:**
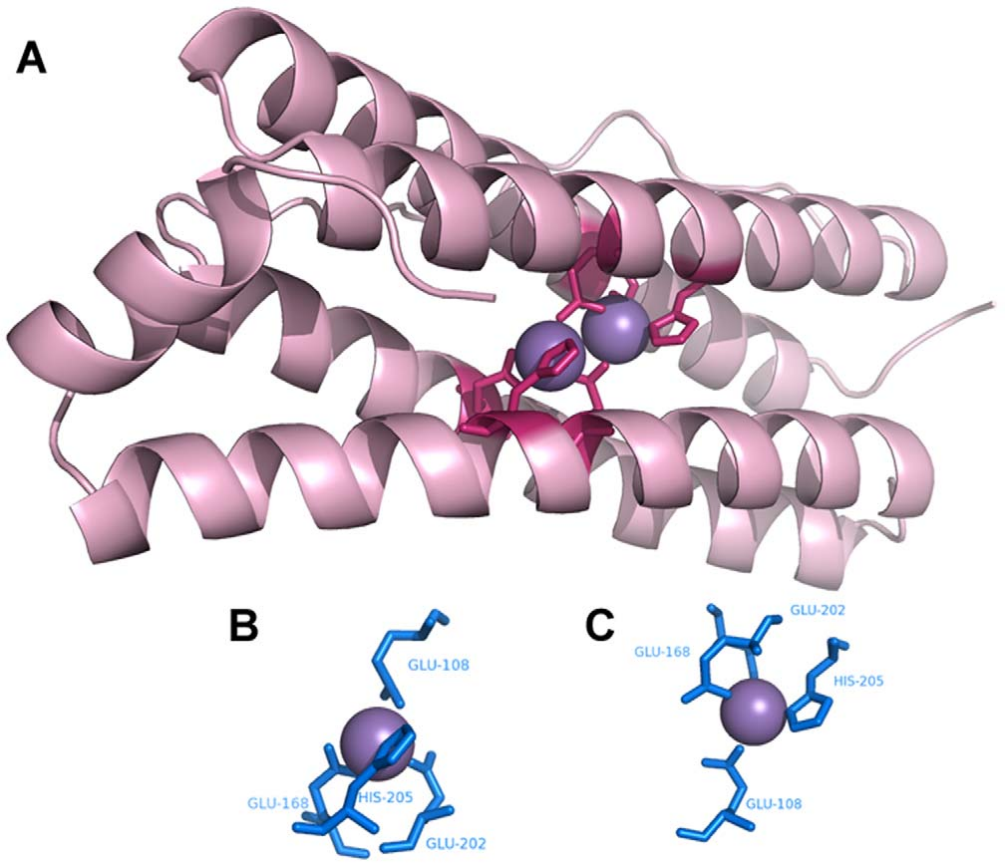
Identification of Mn^2+^–binding sites. **A**. Chain A of cytochrome b1 (PDB ID∶1BFR) as the query protein. **B, C**. Both templates constructed from chain A of ribonucleotide reductase (PDB ID∶1KGP) but oriented differently.

### Performance assessment

The performance of the metal ion–binding site prediction method, i.e., the prediction accuracy (ACC), was defined as the number of true positive and true negative and evaluated using a leave-one-out approach. The accuracy (ACC), the true positive rate (TPR) and false positive rates (FPR) were calculated using the true positive (TP), true negative (TN), false positive (FP), and false negative (FN) values as follows:
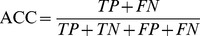
(11)

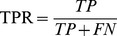
(12)

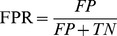
(13)


**Figure 13:**
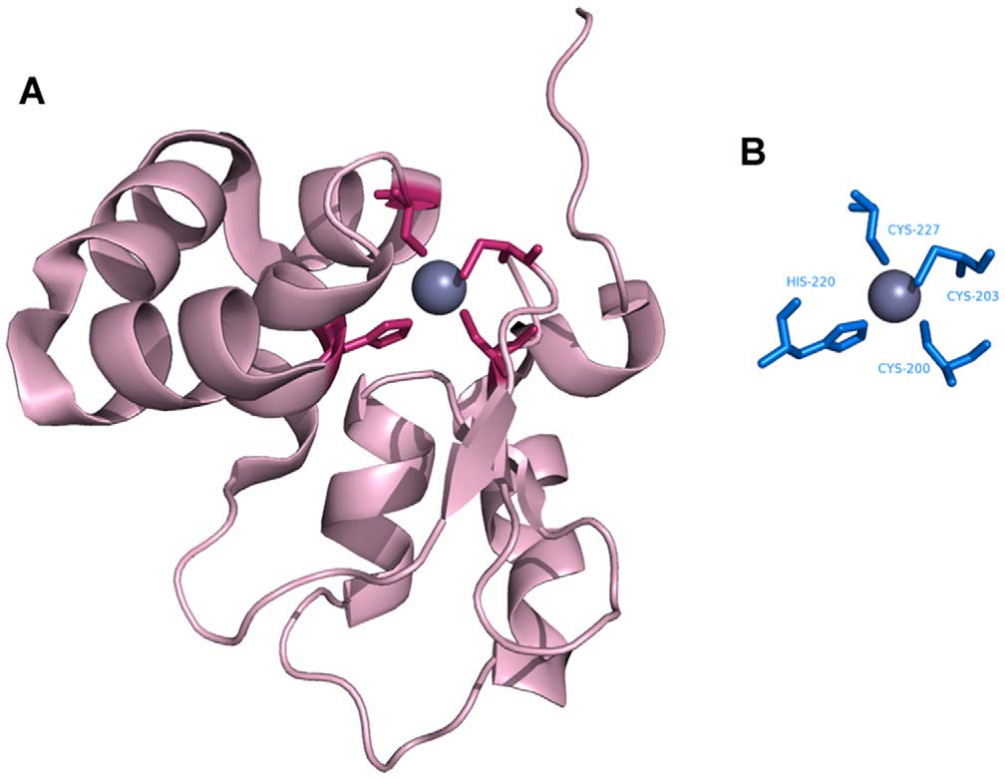
Identification of Zn^2+^–binding sites. **A**. Chain A of DIAP1 (PDB ID∶1JD5) as the query protein. **B**. Template constructed from chain E of the baculoviral IAP repeat–containing protein 4 (PDB ID∶1I3O).

## Results

### Metal ion–binding residue profiles

Spheres each with a 3.5 Å radius from the center of a metal ion were constructed for each metal ion–site in our dataset. We assessed the frequency that each of the 20 amino acids coordinated a metal ion (Fig. 4); those metal ions were found to preferentially bind certain residues, as follows: for Ca^2+^, ASP, GLU, ASN, and GLY; for Cu^2+^, HIS; for Mg^2+^ ASP and GLU; for Fe^3+^, HIS, GLU, ASP, CYS, and TYR; for Mn^2+^, ASP, HIS, and GLU; and for Zn^2+^, CYS and HIS. Notably, each type of metal ion favors specific residues.

The preferred types of atoms surrounding the metal ions are as follows (Figure 5): for Ca^2+^, backbone and side-chain oxygens; for Mg^2+^ and Mn^2+^, side-chain oxygens; for Cu^2+^, Fe^3+^, and Zn^2+^, oxygen, nitrogen, and sulfur. Each metal ion appears to preferentially bind certain atoms in certain residues.

### Predictive performance

For each metal ion, we set the threshold of the normalized metal ion–binding score so that the FPR was ≤5% (Fig. 6). For Ca^2+^–binding proteins, the threshold was 1.6, which gave a 94.1 % accuracy and a TPR of 48.9 %; for Cu^2+^– and Mg^2+^–binding proteins, the threshold was 1.8, which yielded 94.9 % accuracy and a TPR of 85.6 %, and 95.0 % accuracy and a TPR of 61.4 %, respectively; for Fe^2+^– and Mn^2+^–binding proteins, the threshold was 1.0 for 94.9 % accuracy and a TPR of 85.4 %, and 94.6 % accuracy and a TPR of 37.0 %, respectively. The best performance was obtained for Zn^2+^–binding proteins, for which a threshold of 2.2 gave 94.8 % accuracy and a TPR of 71.1 %. The performance of the predictions as a function of the threshold values for six types of metal ion–binding proteins is shown as receiver operating characteristic plot (TPR values vs. FPR values, Fig. 7). The predictive performance was excellent for Cu^2+^– and Fe^3+^–binding proteins and very good for Mn^2+^– and Zn^2+^–binding, but less so for Mg^2+^– and Ca^2+^–binding proteins.

### Comparison with published methods

We compared our results with those obtained using the artificial neural network (ANN) method [30] and the geometric subgraph method [18]. The same types of metal ion–binding sites were used in the three studies, and the methods were each designed to predict every residue within a metal ion–binding protein as a binding or a non-binding residue. When the FPR was ≤ 5 %, our method was more accurate and had greater TPR values than did the ANN method (Table 2). Given the similar accuracies (±1 %), the larger TPR values were especially noticeable for the Cu^2+^– and Fe^3+^–binding proteins (TPR = 85.6 % and 85.4 % for our method, and 36.2 % and 48.8 % for the ANN method, for the two types of proteins, respectively). The TPR values for Mn^2+^ and Zn^2+^ also dramatically improve–from 38.8 % to 61.4 % for Mn^2+^ and from 47.8 % to 71.1 % for Zn^2+^. The TPR for Ca^2+^ also increased from 30.4 % to 48.9 %; however, the improvement was much smaller for Mg^2+^, from 32.4 % to 37.0 %. The average TPR for the six classes of proteins for our study was 60.5 % (FPR≤ 5 %), which is an improvement compared with the results obtained using the geometric subgraph method (TPR, 46.9 %; FPR, 11.9 %).

### Template matching

Figures 8,9,10,11,12,13 show examples of an alignment for each type of metal ion–binding protein and the corresponding template. The structures were drawn by PyMOL [31]. For human cytosolic phospholipase A2 (PDB ID∶ 1RLW; Fig. 8) [32], which has two Ca^2+^–binding sites, seven binding residues were found, all with large normalized metal ion–binding scores. The template that best aligned with the Ca^2+^–binding sites in phospholipase A2 was the chain A of synaptotagmin I C2B-domain (PDB ID∶1K5W) [33] (Fig. 8). The template for the Cu^2+^–binding protein, human ceruloplasmin (PDB ID∶1KCW) [34], almost perfectly aligned with the Cu^2+^–binding site in the A chain of plastocyanin (PDBID∶1BAW) [35] (Fig. 9), although a few FP metal ion–binding residues were also identified. The best predictive performance was found for Fe^3+^–binding proteins. For desulfoferrodoxin (PDB ID∶1DFX) [36], two templates derived from two different proteins, superoxide reductase chain A (PDB ID∶1DO6) [37] and rubrerythrin chain A(PDBID∶1B71) [38], matched its two binding sites, and the nine binding residues, plus an FP, were identified (Fig. 10). Although the identification of Mg^2+^–binding sites was not as successful, because many FPs were associated with high scores, the Mg^2+^–binding site of human mitochondrial deoxyribonucleotidase chain A (PDB ID∶1MH9) [39] was found to be similar to the template constructed from transglutaminase 3 chain B (PDB ID∶1NUG) [40] (Fig. 11). In cytochrome b1 chain A (PDB ID∶1BFR) [41], two Mn^2+^–binding sites were in close proximity and involved the same two glutamic acids (Fig. 12). These binding sites were found using the template from ribonucleotide reductase chain A (PDB ID∶1KGP) [42], even though a reorientation of the template was required during the fragment transformation procedure. For Zn^2+^–binding proteins, a near perfect match was found for chain A of the inhibitor of apoptosis protein DIAP1 (PDB ID∶1JD5) [43] and the template from chain E of the baculoviral IAP repeat-containing protein 4, BIR 2 (PDB ID∶1I3O) [44] (Fig. 13).

## Discussion

In this study, we developed and used a structure comparison method to predict metal ion–binding sites in proteins. During development, we combined conserved structure and sequence information to identify metal ion–binding residues, which are extremely important design elements as they substantially affect the prediction. Our prediction method performed much better for Cu^2+^, Fe,^ 3+^ Mn^2+^, and Zn^2+^ than it did for Ca^2+^ and Mg^2+^, possibly because there are fewer types of residues that bind the transition metal ions compared with those that bind the alkaline earth ions. Thus, the residues and structures of the Ca^2+^– and Mg^2+^–binding sites may be less specific. In particular, we observed that backbone carbonyl oxygens, rather side-chain oxygens, frequently bind Ca^2+^ and Mg^2+^, which indicates that the type of residue is less important–at least for an interaction involving a carbonyl oxygen. Conversely, interactions between backbone atoms and Cu^2+^, Fe^3+^, Mn^2+^, and Zn^2+^ are rare; instead, side-chain atoms bind these ions; causing steric and chemical limitations imposed by the particular side-chain. These two factors, *i.e.*, residue and atom–binding patterns, probably result in smaller sequence alignment scores for the metal ion–binding residues. As such, the final metal ion–binding scores for certain residues may in fact be lower than the threshold value set for metal ion–binding residues.

Our approach yielded excellent predictions for Cu^2+^– and Fe^3+^–binding sites, and very good predictions for Zn^2+^– and Mn^2+^–binding sites. Although the method gave poorer results for Ca^2+^– and Mg^2+^–binding sites, it nonetheless performed better than did the geometric subgraph and ANN methods. Ultimately, for an FPR threshold of 5 % our method achieved an overall 94.6 % accuracy with a TPR of 60.5 %, which is a substantial improvement over other prediction methods currently available. Therefore, our method may find use as a predictor of putative metal ion–binding proteins and their binding. The Linux binary codes for our method are available upon request.

## References

[1] Bernstein FC, Koetzle TF, Williams GJ, Meyer EF, Brice MD (1977). The Protein Data Bank: a computer-based archival file for macromolecular structures.. J Mol Biol.

[2] Tainer JA, Roberts VA, Getzoff ED (1991). Metal-binding sites in proteins.. Curr Opin Biotechnol.

[3] Degtyarenko K (2000). Bioinorganic motifs: towards functional classification of metalloproteins.. Bioinformatics.

[4] Sandier A, Amiel C, Sebille B, Rouchaud JC, Fedoroff M (1997). Chromatographic method involving inductively coupled plasma atomic emission spectrometric detection for the study of metal-protein complexes.. J Chromatogr A.

[5] de la Calle Guntinas MB, Bordin G, Rodriguez AR (2002). Identification, characterization and determination of metal-binding proteins by liquid chromatography. A review.. Anal Bioanal Chem.

[6] Passerini A, Punta M, Ceroni A, Rost B, Frasconi P (2006). Identifying cysteines and histidines in transition-metal-binding sites using support vector machines and neural networks.. Proteins.

[7] Jensen MR, Petersen G, Lauritzen C, Pedersen J, Led JJ (2005). Metal binding sites in proteins: identification and characterization by paramagnetic NMR relaxation.. Biochemistry.

[8] Binet MR, Ma R, McLeod CW, Poole RK (2003). Detection and characterization of zinc- and cadmium-binding proteins in Escherichia coli by gel electrophoresis and laser ablation-inductively coupled plasma-mass spectrometry.. Anal Biochem.

[9] Herald VL, Heazlewood JL, Day DA, Millar AH (2003). Proteomic identification of divalent metal cation binding proteins in plant mitochondria.. FEBS Lett.

[10] Reed GH, Poyner RR (2000). Mn2+ as a probe of divalent metal ion binding and function in enzymes and other proteins.. Met Ions Biol Syst.

[11] Lin HH, Han LY, Zhang HL, Zheng CJ, Xie B (2006). Prediction of the functional class of metal-binding proteins from sequence derived physicochemical properties by support vector machine approach.. BMC Bioinformatics.

[12] Passerini A, Andreini C, Menchetti S, Rosato A, Frasconi P (2007). Predicting zinc binding at the proteome level.. BMC Bioinformatics.

[13] Lin CT, Lin KL, Yang CH, Chung IF, Huang CD (2005). Protein metal binding residue prediction based on neural networks.. Int J Neural Syst.

[14] Schymkowitz JW, Rousseau F, Martins IC, Ferkinghoff-Borg J, Stricher F (2005). Prediction of water and metal binding sites and their affinities by using the Fold-X force field.. Proc Natl Acad Sci U S A.

[15] Shu N, Zhou T, Hovmoller S (2008). Prediction of zinc-binding sites in proteins from sequence.. Bioinformatics.

[16] Levy R, Edelman M, Sobolev V (2009). Prediction of 3D metal binding sites from translated gene sequences based on remote-homology templates.. Proteins.

[17] Deng H, Chen G, Yang W, Yang JJ (2006). Predicting calcium-binding sites in proteins – a graph theory and geometry approach.. Proteins.

[18] Goyal K, Mande SC (2008). Exploiting 3D structural templates for detection of metal-binding sites in protein structures.. Proteins.

[19] Tseng YY, Li WH (2009). Identification of protein functional surfaces by the concept of a split pocket.. Proteins.

[20] Dill KA (1990). Dominant forces in protein folding.. Biochemistry.

[21] Govindarajan S, Goldstein RA (1997). Evolution of model proteins on a foldability landscape.. Proteins.

[22] Parisi G, Echave J (2001). Structural constraints and emergence of sequence patterns in protein evolution.. Mol Biol Evol.

[23] Auld DS (2001). Zinc coordination sphere in biochemical zinc sites.. Biometals.

[24] Golovin A, Dimitropoulos D, Oldfield T, Rachedi A, Henrick K (2005). MSDsite: a database search and retrieval system for the analysis and viewing of bound ligands and active sites.. Proteins.

[25] Lu CH, Lin YS, Chen YC, Yu CS, Chang SY (2006). The fragment transformation method to detect the protein structural motifs.. Proteins.

[26] Henikoff S, Henikoff JG (1992). Amino acid substitution matrices from protein blocks.. Proc Natl Acad Sci U S A.

[27] Murzin AG, Brenner SE, Hubbard T, Chothia C (1995). SCOP: a structural classification of proteins database for the investigation of sequences and structures.. J Mol Biol.

[28] Altschul SF, Gish W, Miller W, Myers EW, Lipman DJ (1990). Basic local alignment search tool.. J Mol Biol.

[29] Gower JC RG (1969). Minimum spanning trees and single-linkage cluster analysis.. Journal of the Royal Statistical Society.

[30] Sodhi JS, Bryson K, McGuffin LJ, Ward JJ, Wernisch L (2004). Predicting metal-binding site residues in low-resolution structural models.. J Mol Biol.

[31] Schrodinger LLC (2010). The PyMOL Molecular Graphics System, Version 1.3r1..

[32] Perisic O, Fong S, Lynch DE, Bycroft M, Williams RL (1998). Crystal structure of a calcium-phospholipid binding domain from cytosolic phospholipase A2.. J Biol Chem.

[33] Fernandez I, Arac D, Ubach J, Gerber SH, Shin O (2001). Three-dimensional structure of the synaptotagmin 1 C2B-domain: synaptotagmin 1 as a phospholipid binding machine.. Neuron.

[34] Zaitseva I, Zaitsev V, Card G, Moshkov K, Bax B (1996). The X-ray structure of human serum ceruloplasmin at 3.1 Å: nature of the copper centres.. Journal of Biological Inorganic Chemistry.

[35] Bond CS, Bendall DS, Freeman HC, Guss JM, Howe CJ (1999). The structure of plastocyanin from the cyanobacterium Phormidium laminosum.. Acta Crystallogr D Biol Crystallogr.

[36] Ana V. Coelho PM, Vilmos Fülöp, Andrew Thompson, A Gonzalez (1997). Desulfoferrodoxin structure determined by MAD phasing and refinement to 1.9-Å resolution reveals a unique combination of a tetrahedral FeS4 centre with a square pyramidal FeSN4 centre.. Journal of Biological Inorganic Chemistry.

[37] Yeh AP, Hu Y, Jenney FE, Adams MW, Rees DC (2000). Structures of the superoxide reductase from Pyrococcus furiosus in the oxidized and reduced states.. Biochemistry.

[38] Sieker LC, Holmes M, Le Trong I, Turley S, Santarsiero BD (1999). Alternative metal-binding sites in rubrerythrin.. Nat Struct Biol.

[39] Rinaldo-Matthis A, Rampazzo C, Reichard P, Bianchi V, Nordlund P (2002). Crystal structure of a human mitochondrial deoxyribonucleotidase.. Nat Struct Biol.

[40] Ahvazi B, Boeshans KM, Idler W, Baxa U, Steinert PM (2003). Roles of calcium ions in the activation and activity of the transglutaminase 3 enzyme.. J Biol Chem.

[41] Dautant A, Meyer JB, Yariv J, Precigoux G, Sweet RM (1998). Structure of a monoclinic crystal from of cyctochrome b1 (Bacterioferritin) from E. coli.. Acta Crystallogr D Biol Crystallogr.

[42] Hogbom M, Huque Y, Sjoberg BM, Nordlund P (2002). Crystal structure of the di-iron/radical protein of ribonucleotide reductase from Corynebacterium ammoniagenes.. Biochemistry.

[43] Wu JW, Cocina AE, Chai J, Hay BA, Shi Y (2001). Structural analysis of a functional DIAP1 fragment bound to grim and hid peptides.. Mol Cell.

[44] Riedl SJ, Renatus M, Schwarzenbacher R, Zhou Q, Sun C (2001). Structural basis for the inhibition of caspase-3 by XIAP.. Cell.

